# Tibial Osteomyelitis Following Prehospital Intraosseous Access

**DOI:** 10.5811/cpcem.2017.9.35256

**Published:** 2017-11-03

**Authors:** Derek Yee, Rahul Deolankar, Jodie Marcantoni, Stephen Y. Liang

**Affiliations:** * Washington University School of Medicine, Division of Medical Education, St. Louis, Missouri; † Washington University School of Medicine, Division of Diagnostic Radiology, St. Louis, Missouri; ‡ Washington University School of Medicine, Division of Emergency Medicine St. Louis, Missouri; § Washington University School of Medicine, Division of Infectious Diseases, St. Louis, Missouri

## Abstract

Intraosseous (IO) access is a lifesaving alternative to peripheral or central venous access in emergency care. However, emergency physicians and prehospital care providers must be aware of the potential for infectious complications associated with this intervention. We describe the case of a HIV-negative, otherwise immunocompetent adult patient who underwent prehospital insertion of a tibial IO device. Following successful resuscitation, the patient developed tibial osteomyelitis requiring multiple operative debridements, soft tissue coverage, and several courses of prolonged antimicrobial therapy. Skin antisepsis prior to device insertion followed by early device removal are important strategies for reducing the risk of infection associated with IO access.

## INTRODUCTION

Intraosseous (IO) access was first described in the 1920s but subsequently fell out of favor due to advances in flexible catheterization.^1^ Over the last two decades, IO access has regained popularity and become increasingly accepted as a mode of rapid vascular access, particularly in prehospital settings, when peripheral venous catheterization cannot be readily obtained. The American Heart Association and the International Liaison Committee on Resuscitation endorse the use of IO access in acute situations as an alternative to intravenous (IV) peripheral access.^2^,^3^ The success rate for IO device insertion is high, with several recent studies demonstrating rates ranging from 80–95%.^4^ Infectious complications related to IO access are rare.^4^ We describe a case in which IO access obtained as part of prehospital care was associated with severe and refractory tibial osteomyelitis in an immunocompetent adult.

## CASE REPORT

A 29-year-old man with a history of depression and heroin dependence, but HIV-negative and otherwise immunocompetent, was found unresponsive with agonal respirations. He was pulseless and cyanotic; cardiopulmonary resuscitation was initiated and the patient was intubated on scene. He was determined to be in ventricular fibrillation and defibrillated several times. Due to poor peripheral vascular access, an IO device was inserted in the left proximal tibia and used to administer naloxone, epinephrine, and amiodarone.

At the local emergency department (ED), advanced cardiac life support continued for ventricular fibrillation alternating with pulseless electrical activity arrest. The patient’s core body temperature registered 28.3°C and aggressive rewarming was undertaken. The IO device was used to administer fluids and medications until it infiltrated and central venous access could be established. After almost 30 minutes, return of spontaneous circulation was achieved. The IO device was removed within an hour of arrival at the local ED.

The patient was subsequently transferred to our tertiary-care hospital ED and admitted to the intensive care unit. He had a complicated hospital course punctuated by volume overload, rhabdomyolysis, and acute kidney injury requiring mechanical ventilation and continuous veno-venous hemodialysis. He also developed a 9×8 cm necrotic wound over the left medial shin at the site of the previous IO device, managed conservatively with topical wound care. He was discharged after two weeks without residual neurological or functional deficits.

**Image 1:**
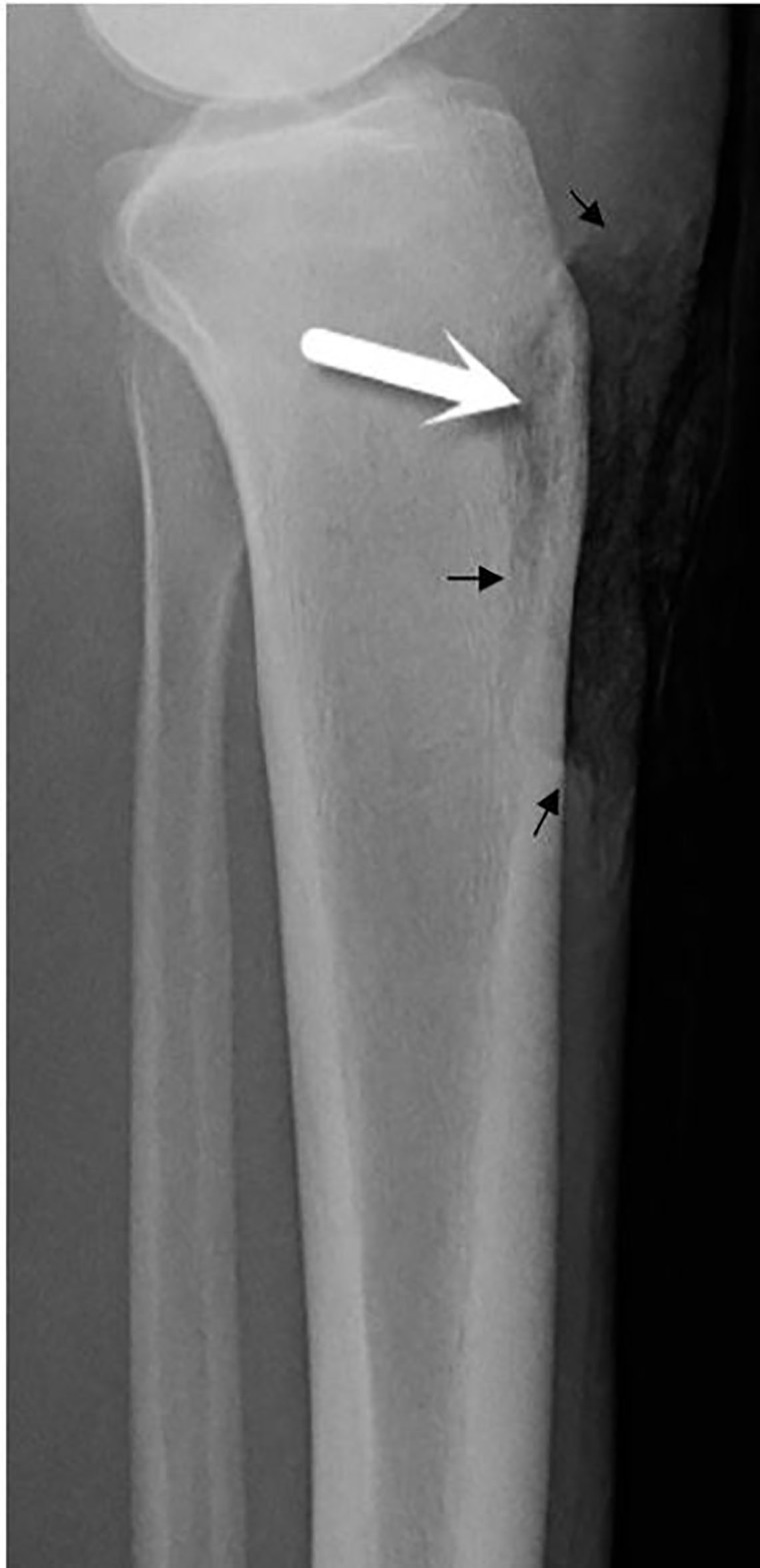
Lateral plain radiography demonstrating a soft tissue defect (black arrows) along the proximal anterior tibia with subtle cortical demineralization (white arrow).

Six weeks later, the patient returned to our ED with increased pain and malodorous serosanguinous drainage from a non-healing 7×5 cm wound involving his left medial shin. Plain radiography demonstrated underlying demineralization of the anterior tibial cortex (Image 1). Operative debridement confirmed necrotic bone and periosteum. Tissue cultures grew *Escherichia coli*, methicillin-sensitive *Staphylococcus aureus* (MSSA), *Bacteroides fragilis*, and other mixed microorganisms, and histopathology was consistent with acute osteomyelitis. Following serial debridement, the patient underwent left medial gastrocnemius muscle flap and split-thickness skin graft coverage of the wound. He was treated with a four-week course of intravenous (IV) ceftriaxone and oral metronidazole followed by two weeks of oral amoxicillin-clavulanic acid. He returned not long after with soft tissue infection involving the muscle flap and received an additional six weeks of intravenous ertapenem due to persistent tibial osteomyelitis.

**CPC-EM Capsule**

What do we already know about this clinical entity?

Osteomyelitis is an infection of the bone that can arise via direct inoculation or hematogenous seeding. Treatment often requires operative debridement and prolonged antimicrobial therapy.

What makes this presentation of disease reportable?

Osteomyelitis due to intraosseous (IO) access is a rare infectious complication that can occur even in immunocompetent adults.

What is the major learning point?

Infection associated with IO access can be severe. Skin antisepsis prior to IO insertion and rapid device removal once alternative access is established can reduce the risk of infection.

How might this improve emergency medicine practice?

Simple infection prevention strategies can help mitigate the risk of infection associated with IO access.

**Image 2:**
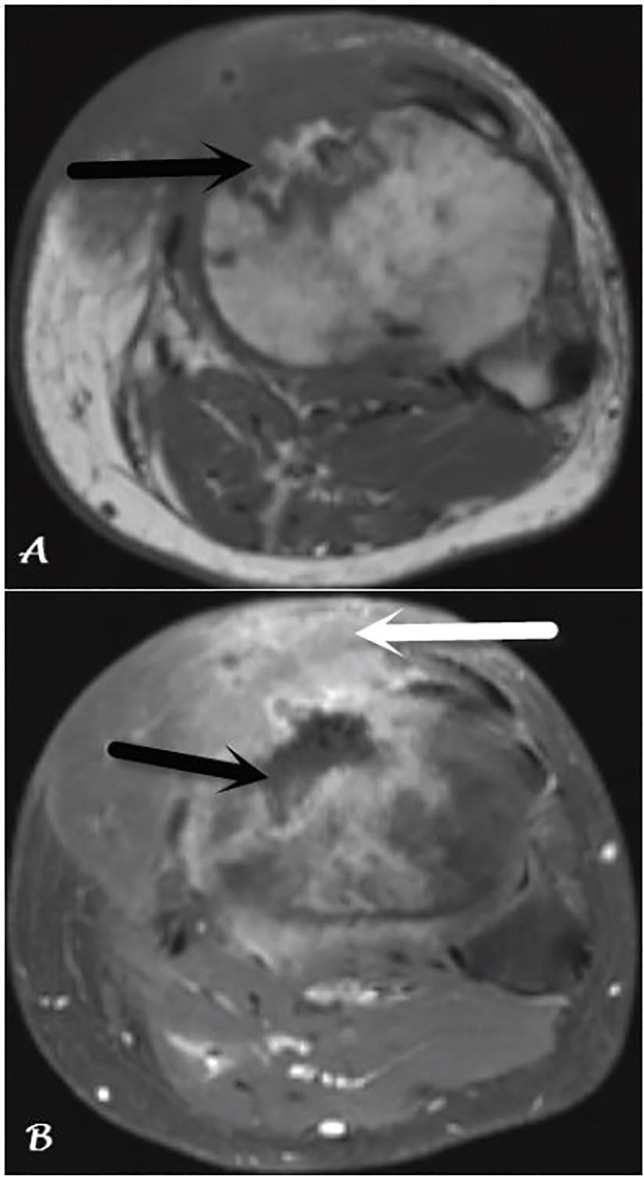
Magnetic resonance imaging of the proximal tibia: A) Axial T1-weighted image demonstrates abnormal heterogeneous marrow signal with associated anterior tibial cortical irregularity (arrow); B) Axial T1-weighted, contrast-enhanced image demonstrates a central region of non-enhancement (black arrow) representing devitalized bone. There is extensive surrounding enhancement of the bone and soft tissue (white arrow). These findings are consistent with osteomyelitis with surrounding soft tissue inflammation.

Several months later, a sinus tract draining purulent material surfaced at the site of his muscle flap. Magnetic resonance imaging demonstrated extensive osteomyelitis of the left proximal tibia with centrally necrotic bone, left knee septic arthritis, and myositis involving the muscle flap (Images 2). The patient subsequently underwent multiple operative incision and debridements of the left tibia with canal reaming and placement of an intramedullary antibiotic drug delivery device. Bone cultures grew MSSA once more. He was treated with six weeks of IV ampicillin-sulbactam and transitioned to chronic antimicrobial suppressive therapy with oral amoxicillin-clavulanic acid. The patient has remained on oral antimicrobials for the past two years with no additional infectious complications involving the left proximal tibia.

## DISCUSSION

IO devices provide rapid vascular access in acute situations when IV access cannot be readily obtained. Battery-powered (EZ-IO™; VidaCare Corporation, San Antonio, TX, USA) and impact-driven (Bone Injection Gun [B.I.G.]™, Waismed, Yokneam, Israel, and FAST1™ Intraosseous Infusion Device (Pyng Medical Corporation, Richmond, BC, Canada) devices provide quick and easy IO access, in addition to traditional manual IO needles.^5^ The most common anatomic location for placement is the proximal tibia owing to the large medullary canal and relative absence of interposing neurovascular structures. Other locations include the distal tibia, femur (particularly in infants and young children), proximal humerus, and the superior sternum.^4^,^6^

While potentially lifesaving, this technique is not without its risks. Complications of IO access can include osteomyelitis, soft tissue infection, skin necrosis, extravasation of infusate, compartment syndrome, tibial fracture, and growth plate injury in pediatric patients. While these complications occur infrequently, with rates reported between 1–5% depending on the device used, they can be serious and life-threatening.^4^,^7^,^8^ Fluid extravasation following IO access is the most frequent complication and can lead to compartment syndrome and tissue necrosis. In contrast, osteomyelitis is rare and sparsely described in the literature. In a review of 1,802 patients, 1,028 of whom were adults, Hallas et al. identified osteomyelitis as a complication in only 0.4% of patients.^9^ Similarly, Rossetti et al. reported a rate of 0.6% in a study of 4,270 pediatric patients.^10^ To date, case reports describing osteomyelitis following IO access have only been described in pediatric patients.^11^–^13^

Osteomyelitis is loosely defined as an infection of the bone. It can arise from direct inoculation of the bone with surgery or trauma, contiguous spread of infection to bone from surrounding tissue, or hematogenous seeding. The most commonly implicated organisms associated with direct inoculation or contiguous spread include skin flora such as *S. aureus* and coagulase-negative staphylococci, as well as aerobic gram-negative bacilli. Other less common organisms can include anaerobes, fungi, enterococci, or mycobacteria.^14^ The gold standard for diagnosis of osteomyelitis is culture of bacteria from bone biopsy obtained under sterile conditions. Treatment often requires operative debridement and IV antimicrobial therapy.

We report a case of severe and refractory tibial osteomyelitis following IO access. The most likely mechanisms were either direct inoculation of the bone from initial insertion of the IO device or contiguous spread to bone following a subclinical or untreated soft tissue infection. Similar to other invasive procedures, sterile technique is paramount when obtaining IO access. Prior to device insertion, the skin should be cleansed with an antiseptic solution such as chlorhexidine. While a direct comparison of chlorhexidine with povidone-iodine prior to bone biopsy or IO device insertion has not been performed, prior studies have demonstrated chlorhexidine-alcohol to be superior in reducing surgical site as well as catheter-associated infections.^15^ It is also important to avoid IO device insertion at a site with active skin and soft tissue infection, whenever possible. Finally, infiltrated IO devices as well as those left *in situ* for greater than 72–96 hours carry an increased risk of infection and should be removed promptly once alternative vascular access can be established.

## CONCLUSION

In conclusion, IO access has become widely accepted as an alternative to venous cannulation in establishing rapid circulatory access in critically ill patients. Adverse events directly related to IO device insertion occur infrequently. With increasing use of IO access in ED and prehospital settings, emergency physicians and prehospital care providers should be cognizant of the risk for serious infectious complications, including osteomyelitis, associated with this intervention and the potential for significant long-term morbidity.
